# Unveiling mitochondrial transfer in tumor immune evasion: mechanisms, challenges, and clinical implications

**DOI:** 10.3389/fimmu.2025.1625814

**Published:** 2025-07-22

**Authors:** Ruoyan Liu, Wenhui Shan, Zhening Wang, Hong Wang, Chunyan Li, Lei Yang, Rui Guo

**Affiliations:** ^1^ Department of Clinical Laboratory, First Affiliated Hospital of Jilin University, Changchun, China; ^2^ Cancer Center, The First Hospital of Jilin University, Changchun, China; ^3^ Paediatrics, The First Hospital of Jilin University, Changchun, China

**Keywords:** mitochondrial transfer, tumor immune evasion, tumor microenvironment, immunology, bibliometric analysis

## Abstract

**Background:**

Mitochondrial transfer, the intercellular transmission of mitochondria via tunneling nanotubes(TNTs), extracellular vesicles(Evs), or cell fusion, has emerged as a critical mechanism in cancer progression. Increasing evidence suggests that this phenomenon not only supports tumor cell metabolism and drug resistance but also contributes to immune evasion, a hallmark of cancer.

**Objective:**

This study aims to systematically explore the intellectual structure, research hotspots, and emerging trends of mitochondrial transfer in tumor immune evasion using bibliometric and visualization tools.

**Method:**

Publications from 2003 to 2025 were retrieved from the Web of Science Core Collection. CiteSpace and VOSviewer were used to analyze annual outputs, co-occurring keywords, citation bursts, clustering patterns, and co-cited references.

**Results:**

A total of 124 records were analyzed. The number of publications increased sharply after 2017, indicating growing research interest. Key terms such as “tunneling nanotubes,” “mitochondrial transfer,” and “immune escape” were frequently co-mentioned. Although “immune escape” is retained here to reflect the exact terminology used in the bibliometric database (Web of Science Core Collection), the manuscript text consistently adopts the term “immune evasion” for conceptual clarity and terminological standardization. Timeline cluster analysis identified several sustained hotspots, including acute myeloid leukemia (AML), stromal cells, and the cancer microenvironment. Citation burst analysis revealed emerging attention toward “expression,” “stem cells,” and “tumor microenvironment” in recent years.

**Conclusion:**

Mitochondrial transfer has transitioned from a structural phenomenon to a key immunological modulator in cancer. This bibliometric analysis highlights its central role in immune evasion and identifies future research directions for therapeutic exploitation.

## 1 Introduction

Tumor immune evasion is a critical hallmark of cancer, enabling malignant cells to escape immune surveillance and resist immunotherapeutic interventions (1). Recent research has revealed that this escape is not solely governed by immune checkpoint regulation or antigen presentation failure, but also by dynamic intercellular communication that modulates immune cell function (2).

Among these mechanisms, mitochondrial transfer has emerged as a novel and underexplored pathway. This phenomenon refers to the direct intercellular movement of mitochondria, most commonly via TNTs (3, 4), EVs (5), or cell fusion (6) Initially reported in tissue regeneration and stem cell rescue contexts, mitochondrial transfer is now increasingly linked to cancer progression, drug resistance, and importantly, immune evasion (7, 8). Recent evidence indicates that tumor-derived EVs can deliver mitochondrial components, such as mitochondrial DNA and proteins, to recipient cells within the tumor microenvironment. Guan et al. provided a comprehensive review showing that this mitochondrial cargo reprograms the metabolism of recipient cells and contributes to immune evasion (4). Moreover, mitochondrial transfer from mesenchymal stromal cells(MSCs) to CD8^+^ T cells has been shown to suppress T cell proliferation and IFN-γ production by downregulating transcription factors such as T-bet and Eomes, thereby impairing cytotoxic function (9). Conversely, tumor cells acquiring mitochondria from T cells or stromal cells gain enhanced metabolic fitness, which in turn promotes T cell exhaustion and facilitates immune evasion (10).Recent studies have demonstrated that tumor cells can directly transfer mitochondria to immune cells, such as CD8^+^ T lymphocytes, leading to metabolic reprogramming and subsequent functional exhaustion, thereby impairing cytotoxicity. In particular, Zhang et al. used a combination of *in vivo* tumor models and co-culture experiments to demonstrate mitochondrial acquisition by CD8^+^ T cells. This uptake coincided with IFN-γ downregulation and increased programmed cell death protein 1(PD-1) and thymocyte selection-associated HMG box(TOX) expression, suggesting mitochondrial transfer contributes to T cell functional exhaustion. However, whether blocking transfer can reverse exhaustion remains unclear (10). Similarly, Marlein et al. showed that AMLblasts utilize NADPH oxidase-mediated signaling to trigger mitochondrial transfer from bone marrow stromal cells, decreasing reactive oxygen species stress and blunting immunogenicity (11). Independent work by Moschoi et al. demonstrated that bone marrow stromal cells transfer mitochondria to AML blasts via tunneling nanotubes and macropinocytosis, promoting leukemic survival and chemotherapy resistance (12). Together with Marlein’s study, these findings emphasize the role of mitochondrial donation in hematological tumor fitness.

These findings underscore the dual role of mitochondrial transfer as both a metabolic modulator and an immune reprogramming tool within the tumor microenvironment (TME) (10).

Bibliometric analysis offers a quantitative and visual approach to map the intellectual landscape of this research field. Tools like CiteSpace and VOSviewer have been widely used to identify knowledge structures and predict emerging trends in biomedical domains (13).

Mitochondrial transfer has been widely documented in multiple tumor types, including AML, glioma, and breast cancer, across diverse interaction modes such as tumor–stromal, tumor–immune, and tumor–tumor communication (4). Guan et al. comprehensively reviewed the functional roles of mitochondrial transfer in cancer aggressiveness, immune suppression, and resistance to therapy, supporting its relevance across solid and hematologic malignancies (4). Recent studies also suggest that mitochondrial transfer may activate innate immune sensing pathways, such as the STING pathway, thereby contributing to antitumor immunity in certain contexts (14). Here, we conducted a comprehensive bibliometric analysis of publications from 2003 to 2025 to explore the developmental trajectory of mitochondrial transfer research in cancer immunity. By mapping co-occurrence patterns, citation bursts, author collaborations, and high-impact references, this study aims to provide a panoramic understanding of the field and inform future translational and therapeutic strategies.

## 2 Materials and methods

This bibliometric study was based on data retrieved from the Web of Science Core Collection (WoSCC), one of the most comprehensive and standardized databases for scientific literature analysis. The search was conducted on April 28, 2025, using the following strategy: TS=(“mitochondrial transfer” OR “intercellular mitochondria” OR “tunneling nanotube*” OR MIRO1 OR TNFAIP2) AND TS=(cancer OR tumor* OR carcinoma OR neoplasm) AND TS=(immune* OR lymphocyte* OR “T cell*” OR “CD8*” OR “immune escape” OR exhaustion OR “checkpoint blockade” OR PD-1 OR PD1 OR CTLA-4). The TS (Topic Search) field in WoSCC includes terms from titles, abstracts, author keywords, and Keywords Plus. This query was designed to maximize specificity toward mitochondrial trafficking with immunological relevance. Broader terms such as “extracellular vesicles” or “organelle transfer” were tested but yielded a high proportion of unrelated entries. This strategy may have excluded literature involving indirect mechanisms or indexed in other databases (e.g., PubMed, Scopus), a limitation discussed in Section 4.4.No language restrictions were applied, and the time span was set from 2003 to 2025. A total of 124 records were retrieved and exported in plain text and Excel formats for further analysis.

For visual analysis and mapping, we used CiteSpace (version 6.4.R1, Chaomei Chen Lab) and VOSviewer (version 1.6.20). CiteSpace was employed to construct keyword co-occurrence networks, perform timeline clustering, detect citation bursts, and analyze co-cited references. The g-index (k = 25) was selected to balance between citation depth and visualization clarity. Trial runs using k = 15 and k = 50 were conducted; the former excluded relevant moderate-impact papers, while the latter produced dense and unreadable network maps.

VOSviewer was used primarily to visualize keyword clusters and author collaboration networks based on co-authorship data. The software’s mapping layout enabled intuitive identification of thematic clusters and node density. In addition, Microsoft Excel was used to generate the annual publication and citation trend plots.

All data used in this study were obtained from public sources and did not involve human participants, ethical approval, or confidential patient information.

## 3 Results

### 3.1 Annual publication trend

**Figure 1:**
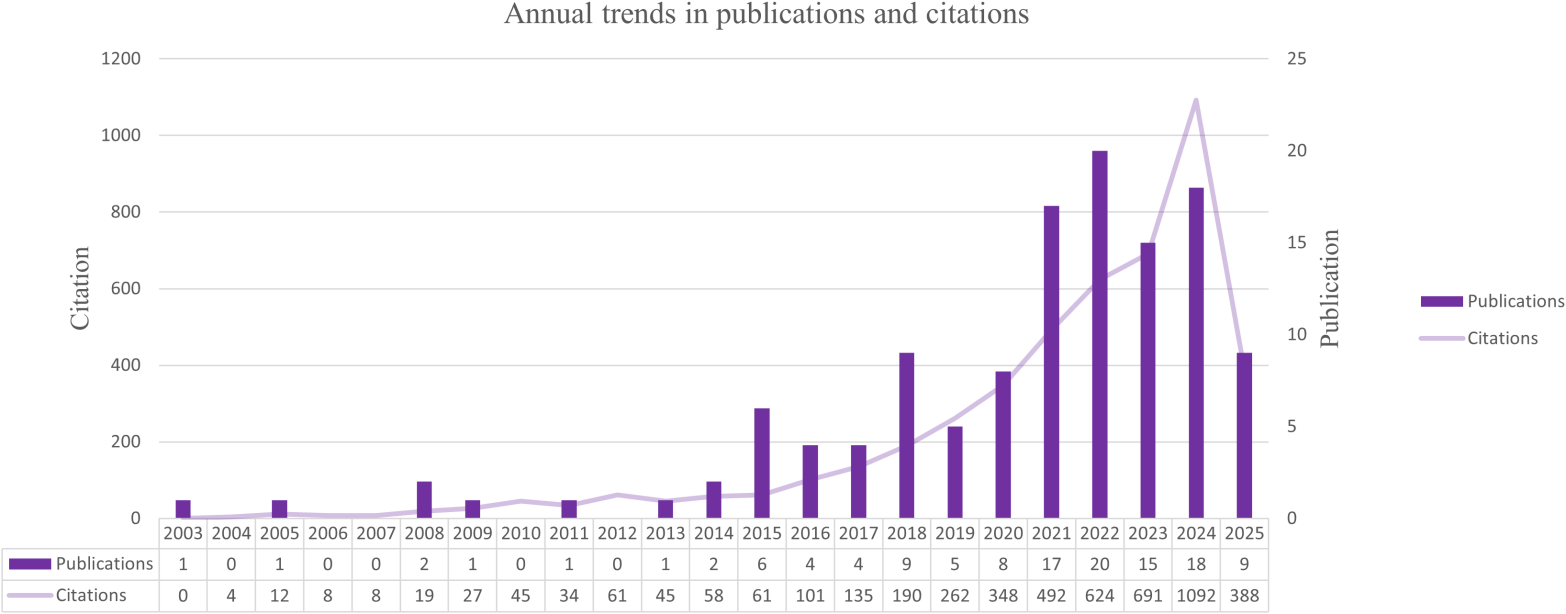
Annual trends in publications and citations related to mitochondrial transfer in tumor immunity from 2003 to 2025. A sharp rise in publication volume and citation frequency after 2020 indicates accelerated research activity in this field.

To characterize the overall development of research on mitochondrial transfer in tumor immune evasion, we analyzed the annual number of publications and their citation frequency from 2003 to 2025. As illustrated in **Figure 1**, this field experienced a relatively stagnant phase during the early years (2003–2015), with fewer than 10 papers published annually.

Beginning around 2016, the number of publications began to grow steadily, followed by a marked surge after 2020. A peak was observed in 2023, with 124 articles published. This uptick reflects the expanding scientific interest in the role of mitochondrial transfer as a modulator of immune responses in cancer. In parallel, the annual citation counts increased sharply, indicating rising scholarly attention and growing influence of foundational studies in this area.

This temporal pattern reflects a growing research interest in the immunomodulatory roles of mitochondrial transfer in cancer, as further discussed in the mechanistic section (Discussion 4.1).

### 3.2 Keyword co-occurrence and clustering

**Figure 2:**
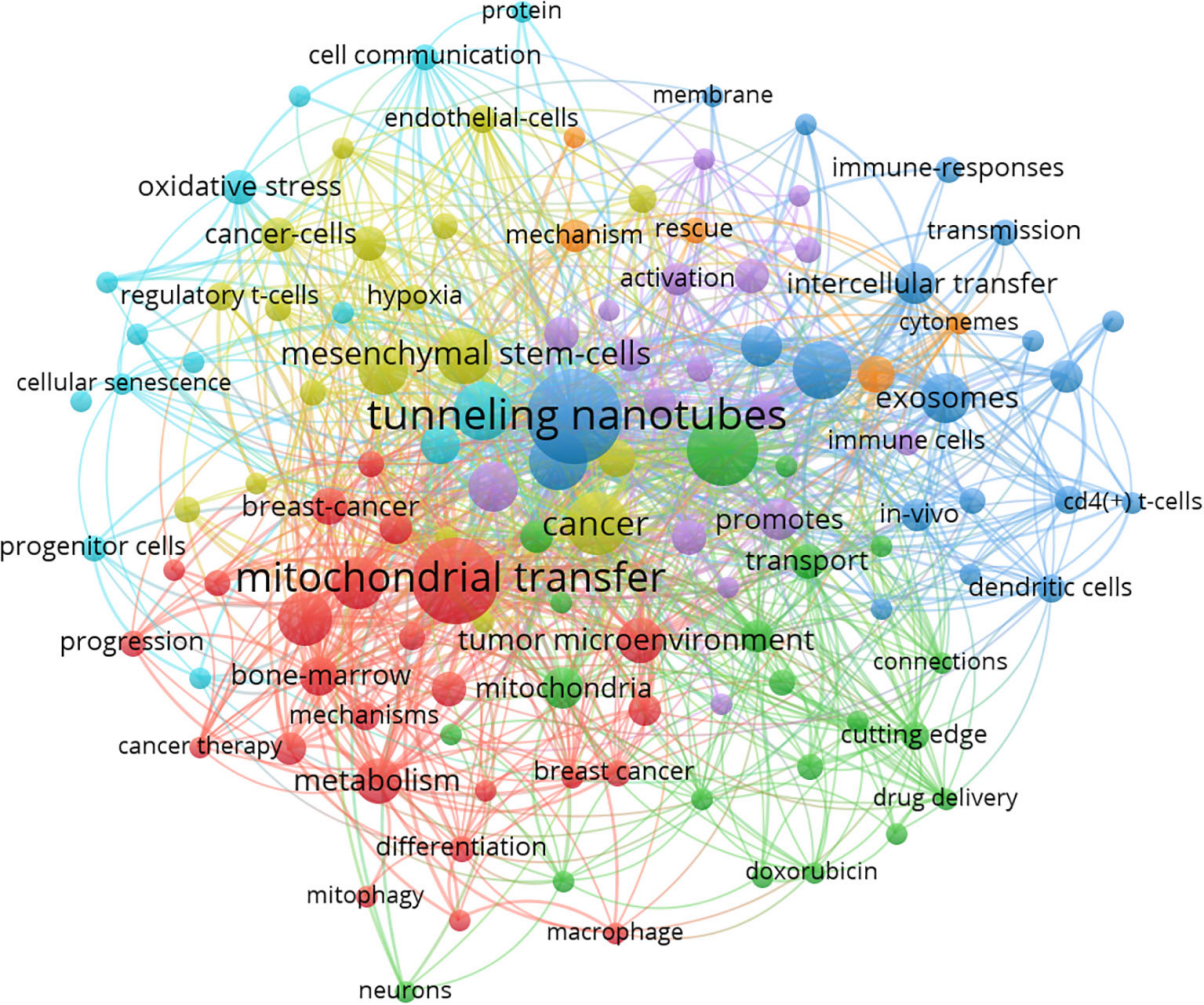
Co-occurrence network of author keywords generated by VOSviewer (threshold ≥3 occurrences). Node size indicates keyword frequency; edge thickness reflects co-occurrence strength. Colors represent thematic clusters automatically assigned by VOSviewer. The red cluster centers on “mitochondrial transfer”, “metabolism”, and “bone marrow”, reflecting metabolic and structural aspects. The green cluster includes terms such as “macrophage”, “mitophagy”, and “drug delivery”, suggesting immunometabolic regulation. The blue cluster features “intercellular transfer”, “exosomes”, and “immune responses”, emphasizing intercellular communication in the tumor immune microenvironment. Modularity Q = 0.71; silhouette score = 0.65.

To identify the main research themes and conceptual structure of this field, we analyzed the co-occurrence patterns of author keywords using VOSviewer. A total of 918 unique keywords were extracted from the dataset. After setting a minimum occurrence threshold of 3, 120 keywords met the inclusion criteria and were visualized in a network map (**Figure 2**). Higher thresholds such as 5 or 10 were tested but failed to retain emerging or low-frequency terms crucial to mechanistic trends. Thus, 3 was retained to preserve thematic inclusivity.

The resulting co-occurrence network revealed several tightly clustered groups, each representing a distinct thematic focus. The largest cluster (red) centers on “tunneling nanotubes,” “intercellular mitochondrial transfer,” and “immune escape,” reflecting the core biological mechanism by which mitochondria are transferred and its immunological consequences. The green cluster includes terms like “mesenchymal stem cells,” “tumor microenvironment,” and “stromal cells,” suggesting a focus on the donor-recipient cell interaction context. Other clusters highlight related topics such as oxidative stress, reactive oxygen species regulation, macrophage polarization, and PD-1 signaling, indicating the expanding scope of the field into immune checkpoint modulation and metabolic crosstalk.

Node size in the map corresponds to the frequency of each keyword, while link strength indicates the co-occurrence strength between terms. The keyword “mitochondrial transfer” appeared most frequently, confirming its centrality, while keywords like “CD8+ T cell,” “exhaustion,” and “metabolic reprogramming” suggest increasing emphasis on immunological endpoints.

Together, these clusters reflect the interdisciplinary nature of mitochondrial transfer research, encompassing cancer metabolism, immunology, cell communication, and therapeutic development.

### 3.3 Analysis of cited references

**Figure 3:**
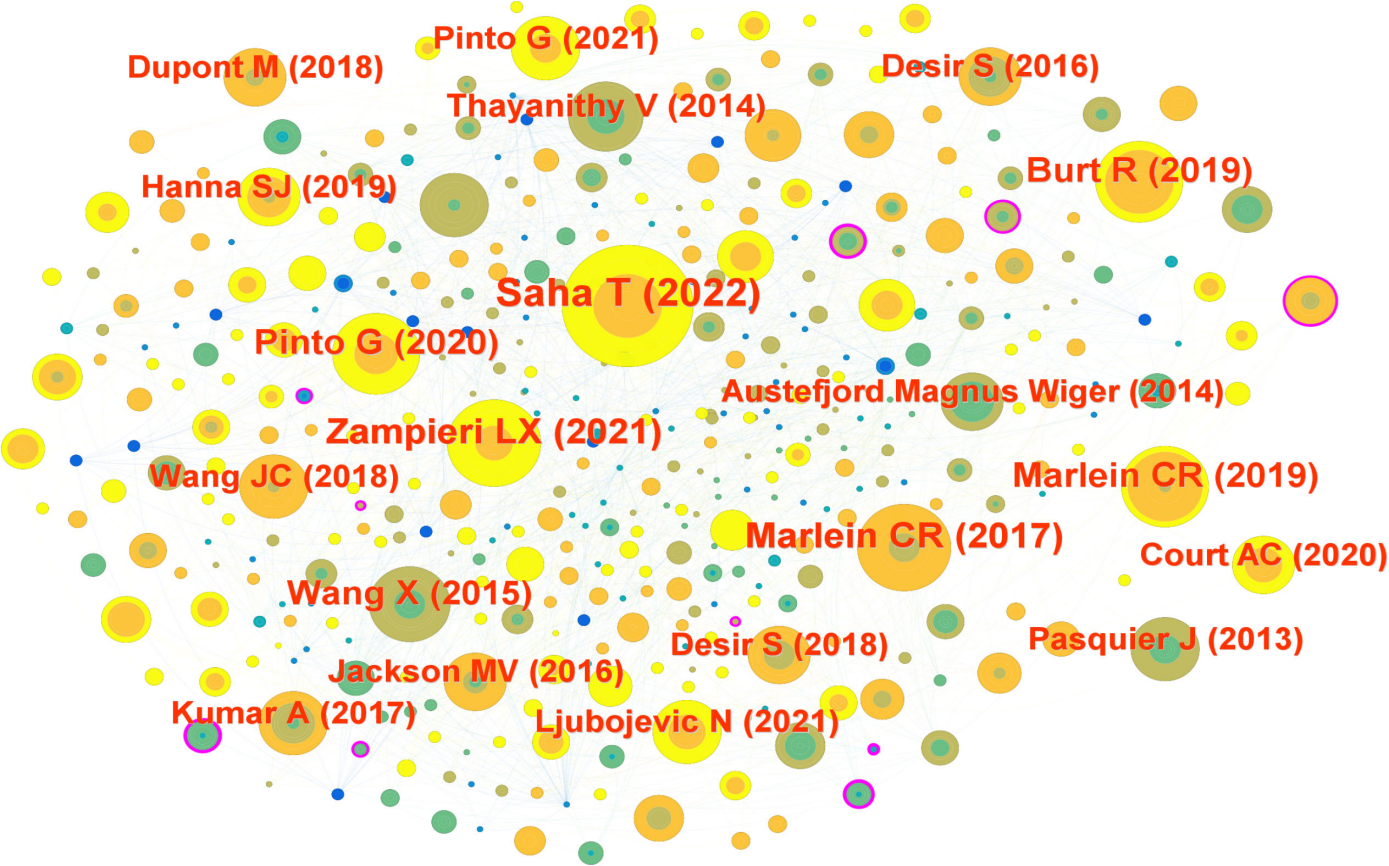
Reference co-citation network visualized using CiteSpace. Each node represents a cited article; node size corresponds to citation frequency, and node color indicates the average publication year. Clustering was performed using Pathfinder and pruning algorithms. Major clusters include foundational studies such as Saha et al. (2022), Marlein et al. (11), and Pasquier et al. (2013). Modularity Q = 0.74; silhouette score = 0.72.

To elucidate the foundational knowledge base of mitochondrial transfer in tumor immunity, we performed a co-cited reference analysis using CiteSpace. The resulting network contained 656 nodes and 1,576 links, representing pivotal literature and their co-citation relationships. Node size reflects citation frequency, while node color corresponds to the average publication year, highlighting temporal trends in intellectual influence (**Figure 3**).

The most highly cited reference was by Saha et al. (15), published in Nature Nanotechnology, which demonstrated that tumor-derived mitochondria can be transferred into CD8^+^ T cells, impairing their cytotoxic capacity. Other frequently co-cited references included Zampieri et al. (16) (Int J Mol Sci, 15 citations), Marlein et al. (11, 17) (Blood and Cancer Research, 14 citations), and Pinto et al. (18) (Trends in Cancer, 14 citations). These studies collectively revealed that mitochondrial trafficking supports tumor survival, redox adaptation, and immune evasion under metabolic stress.

Several older but still central works, such as Pasquier et al. (19) on cell fusion-mediated organelle transfer and Thayanithy et al. (20) on TNT-mediated mitochondrial exchange, continue to serve as theoretical anchors in the field.

Interestingly, many of the most influential articles were published between 2017 and 2023, indicating a recent expansion of interest in the immunological consequences of mitochondrial transfer. This temporal clustering suggests that the field is transitioning from descriptive observations to mechanistic and translational research.

The co-citation map also revealed tightly connected sub-networks, such as those centered on oxidative stress, bone marrow stromal support, and T cell exhaustion, indicating the interdisciplinary convergence of mitochondrial biology, immunometabolism, and cancer therapy.

### 3.4 Citation burst keywords

**Figure 4:**
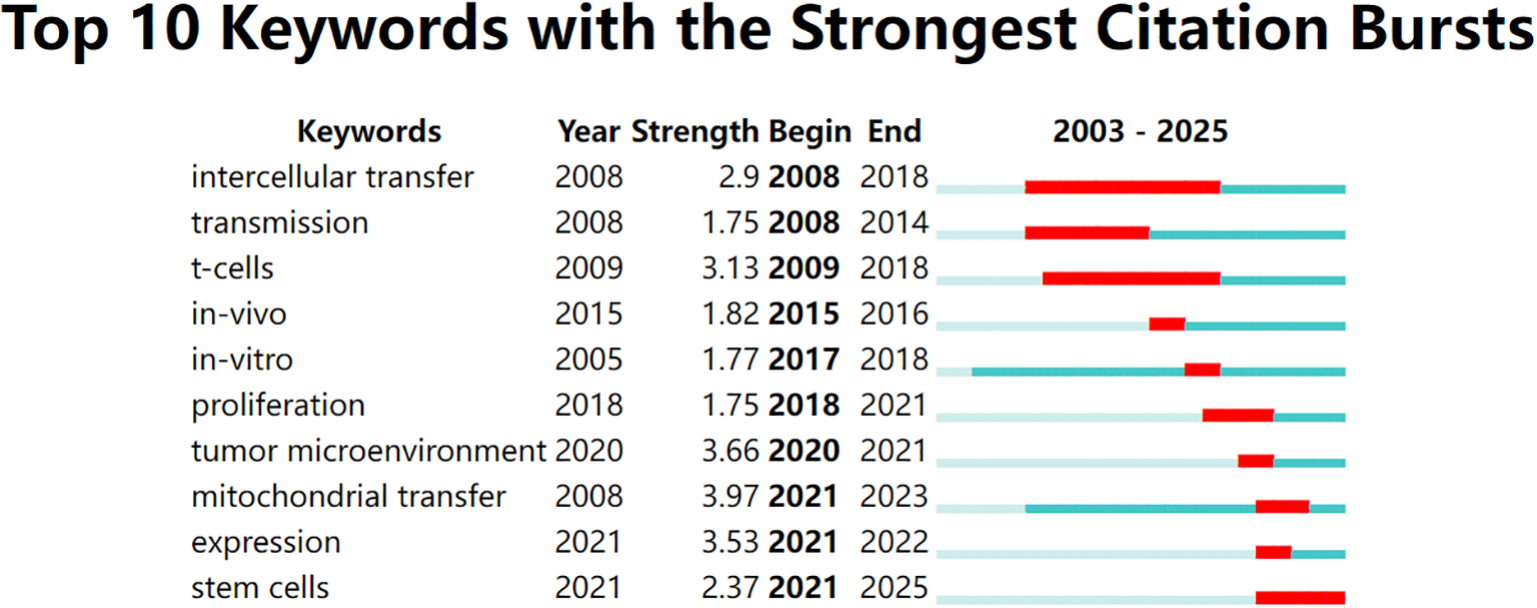
Top 10 keywords with the strongest citation bursts from 2003 to 2025, identified using CiteSpace. Red bars indicate the time periods during which each keyword experienced a burst in citations, while the full horizontal line represents the overall time span. Recent bursts in keywords such as “mitochondrial transfer”, “tumor microenvironment”, and “expression” (beginning after 2020) reflect emerging research interest in mitochondrial dynamics and immunometabolic regulation.

To identify emerging hotspots, we conducted a citation burst analysis of keywords. This method highlights terms that received significant attention during a defined period, often reflecting shifting research frontiers. As shown in **Figure 4**, early burst terms such as intercellular transfer, transmission, and Tcells emerged around 2008–2009, representing foundational interest in intercellular communication and immune regulation.

**Figure 5:**
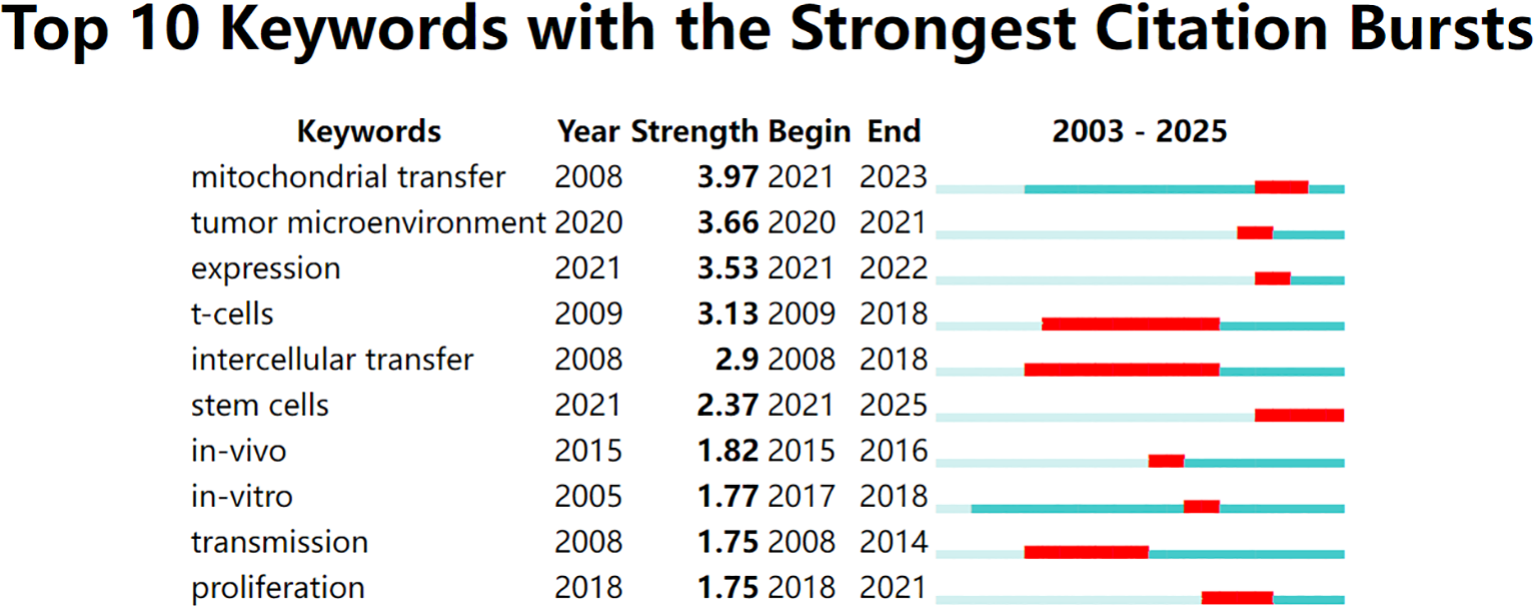
Top 10 keywords with the strongest citation bursts between 2003 and 2025, visualized using CiteSpace. The red bars indicate the time periods during which each keyword experienced a significant increase in citations (i.e., burst periods), while the blue-green bars represent the full timespan of analysis. Notably, “mitochondrial transfer,” “tumor microenvironment,” and “expression” have shown the strongest recent bursts, reflecting growing research attention toward immunometabolic mechanisms of tumor progression in recent years.

In recent years, a different set of keywords has shown strong citation bursts. As shown in **Figure 5**, terms like mitochondrial transfer, TME, and expression became highly cited after 2020. Among these, mitochondrial transfer had the highest burst strength (3.97), indicating intensified research focus between 2021 and 2023. Other notable recent terms include stem cells and immune evasion, pointing to a growing interest in the immunometabolic functions of mitochondrial exchange. Overall, five of the top ten keywords began their bursts after 2020, suggesting that this field is undergoing a rapid conceptual evolution.

### 3.5 Cluster analysis

**Figure 6:**
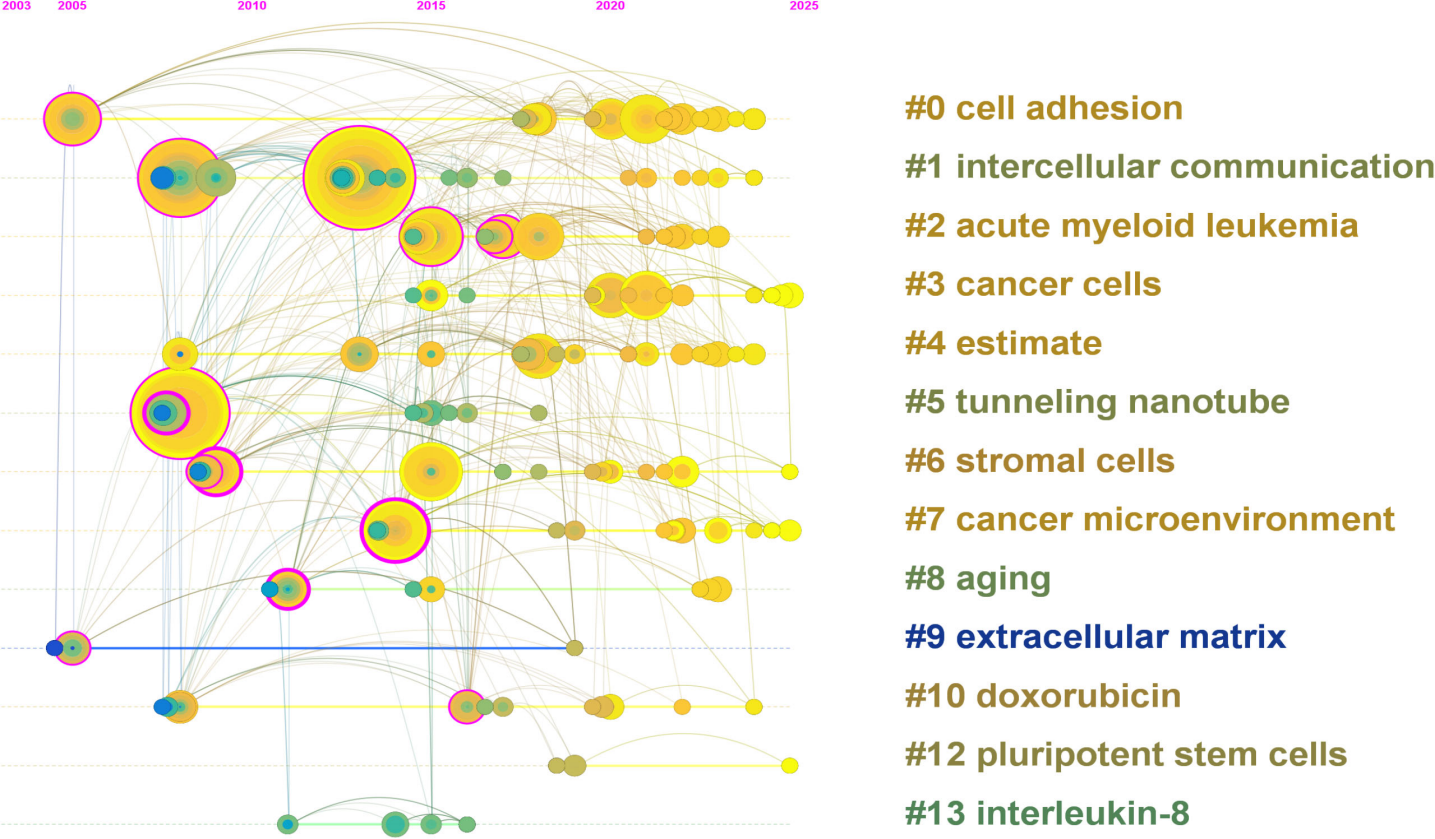
Timeline view of keyword clustering generated by CiteSpace (2003–2025). Each horizontal line represents a distinct keyword cluster, with nodes indicating high-frequency terms and their citation activity over time. Node size reflects citation frequency, and the color gradient (green to yellow) indicates the temporal distribution of research activity. Early clusters such as “cell adhesion” (#0), “intercellular communication” (#1), and “cancer cells” (#3) dominated initial research stages. In contrast, recent clusters including “cancer microenvironment” (#7), “stromal cells” (#6), and “tunneling nanotube” (#5) reflect growing attention to immune interactions and mitochondrial dynamics. Emerging topics such as “extracellular matrix” (#9) and “pluripotent stem cells” (#12) indicate expanding interest in microenvironmental regulation and regenerative approaches in oncology.

To better understand the structural framework and developmental trajectory of mitochondrial transfer in tumor immune evasion, we conducted a clustering analysis using CiteSpace. The timeline view (**Figure 6**) illustrates the evolution of major thematic clusters over time. Each horizontal line represents a specific cluster, and the nodes on the line indicate keywords with strong citation links within that cluster.

A total of 14 meaningful clusters were generated, with the largest including “cell adhesion” (#0), “intercellular communication” (#1), “acute myeloid leukemia” (#2), and “cancer cells” (#3). Cluster #5 (“tunneling nanotube”) and cluster #6 (“stromal cells”) reflect mechanistic components of mitochondrial transfer, while clusters such as #7 (“cancer microenvironment”) and #9 (“extracellular matrix”) suggest increasing emphasis on the immune-regulatory aspects of the TME.

The timeline distribution reveals that early research efforts (2003–2015) concentrated on fundamental cell biology terms such as “cell adhesion” and “communication,” while recent years (post-2019) have witnessed the rise of immunologically relevant topics including “immune response,” “microenvironment,” and “pluripotent stem cells.” Notably, clusters such as “tunneling nanotube” and “extracellular matrix” have maintained sustained activity across multiple time periods, suggesting their central role in the field’s development.

This cluster map underscores the evolution from basic mitochondrial biology to more specialized discussions of immunological interactions and therapeutic implications in the tumor context. While the bibliometric analysis provides an overview of research activity and thematic evolution, the interpretation of these clusters requires integration with mechanistic insights from primary studies. Therefore, we next discuss how the identified trends—such as increased focus on CD8^+^ T cell exhaustion and stromal cell interactions—may reflect deeper immunological processes underpinning tumor resistance.

## 4 Discussion

### 4.1 Mechanisms of mitochondrial transfer and immune modulation

Since 2020, several studies have linked mitochondrial transfer to critical immunological mechanisms, including metabolic suppression, T cell dysfunction under hypoxia, and exhaustion phenotype (e.g., PD-1, TOX expression), reinforcing the biological relevance of the clusters identified in our bibliometric analysis. Mitochondrial transfer, originally characterized in the context of stem cell rescue and tissue regeneration, has emerged as a pivotal mechanism in shaping tumor immune evasion. This growing prominence is clearly reflected in our bibliometric analysis, which revealed that the number of publications in this field increased sharply after 2017, indicating rapidly growing research interest. The capacity of tumor cells to hijack metabolic and organelle resources from stromal or immune cells enables them to establish a more aggressive and therapy-resistant phenotype. This process occurs primarily through TNTs, EVs, or direct cell fusion, facilitating the exchange of intact mitochondria across cell boundaries. Indeed, our analysis of co-occurring keywords identified “tunneling nanotubes,” “mitochondrial transfer,” and “immune escape” as frequently co-mentioned key terms, underscoring that this exchange mechanism and its immunological consequences are central to current research.

Recent single-cell studies have provided direct evidence that tumor cells can donate mitochondria to infiltrating T cells, leading to metabolic rewiring and exhaustion of their effector function. For instance, Zhang et al. showed that mitochondrial acquisition by CD8^+^ T cells drives a dysfunctional phenotype and reduces cytokine secretion, correlating with poor therapeutic outcomes (10). This was accompanied by increased expression of immune checkpoint receptors such as PD-1 and TIM-3, which are markers of terminal exhaustion. These findings reposition mitochondrial transfer as not merely a bystander event, but as a driver of immune suppression in the TME. Building on these findings, mitochondrial transfer is increasingly recognized as a contributor to immune dysfunction in the TME. Particularly, recent mechanistic studies have linked mitochondrial donation from tumor or stromal cells to T cell exhaustion and immune suppression, supporting the idea that mitochondrial dynamics modulate immunotherapeutic outcomes. These observations suggest a hypothetical model wherein mitochondrial transfer in hypoxic, immune-infiltrated tumors induces CD8^+^ T cell metabolic dysfunction and exhaustion. This, in turn, may contribute to resistance to immune checkpoint blockade therapies. While further validation is needed, this model provides a conceptual framework linking metabolic stress, mitochondrial dynamics, and immune evasion.

### 4.2 Metabolic adaptation and tumor survival

The metabolic consequences of mitochondrial donation are profound. Transferred mitochondria can enhance oxidative phosphorylation, elevate ATP production, and reduce reactive oxygen species stress in recipient tumor cells, thereby promoting survival under immune or therapeutic pressure (8). In AML, bone marrow stromal cells supply mitochondria to leukemic blasts via NOX2-mediated pathways, conferring resistance to oxidative damage and chemotherapy (11).

From an immunological perspective, mitochondria serve as signaling organelles that regulate innate and adaptive immune responses via DAMPs, mitochondrial DNA (mtDNA), and reactive oxygen species. The abnormal trafficking of mitochondria may disrupt immune sensing by antigen-presenting cells or alter the cytokine landscape. Our bibliometric study concluded that mitochondrial transfer has transitioned from a structural phenomenon to a key immunological modulator in cancer, highlighting its critical role in these complex immune interactions within the TME. Some studies have observed that mitochondrial transfer can modulate macrophage polarization, enhance M2-like features, and suppress antigen presentation (9).

### 4.3 Heterogeneity and immune context dependence

Our bibliometric analysis identified several sustained research hotspots, notably including AML, stromal cells, and the cancer microenvironment, suggesting focused research efforts in these areas. Although mitochondrial transfer is increasingly reported in leukemia and breast cancer, its roles in other tumors with high T cell infiltration like melanoma, non-small cell lung cancer, and renal carcinoma remain underexplored. While our bibliometric data points to current hotspots, it implicitly suggests that a detailed exploration of mitochondrial transfer mechanisms in these other key immunotherapy-responsive cancers may represent a significant knowledge gap and an opportunity for future investigation. These cancers exhibit high levels of T cell infiltration and immune checkpoint blockade (ICB) sensitivity, yet paradoxically harbor populations of exhausted or non-responsive TILs. It is plausible that mitochondrial exchange between tumor and immune cells contributes to this phenomenon by altering the metabolic landscape of TILs.

Recent studies have highlighted the metabolic vulnerability introduced by mitochondrial acquisition in T cells. For instance, Zhang et al. demonstrated that mitochondrial uptake by CD8^+^ T cells reprograms their metabolic state, shifting from glycolysis to oxidative phosphorylation, which diminishes their proliferative capacity and effector function in the TME (10). This metabolic rewiring not only depletes T cells of critical bioenergetic resources but also induces an exhaustion-like phenotype, marked by the upregulation of inhibitory receptors such as PD-1 and TIM-3 (T cell immunoglobulin and mucin-domain containing protein 3) and decreased cytokine production. These findings suggest that mitochondrial transfer may serve as a mechanism of immune evasion in solid tumors with high immune infiltration.

Despite its emerging relevance, the study of mitochondrial transfer in tumors with high T cell infiltration like melanoma, non-small cell lung cancer, and renal carcinoma remains sparse. These tumors, characterized by substantial T cell infiltration, frequently exhibit immune checkpoint resistance and therapeutic failure, potentially linked to mitochondrial-mediated metabolic suppression of TILs. In the context of hypoxic TME, which are common in non-small cell lung cancer and renal carcinoma, mitochondrial transfer is hypothesized to exacerbate immune suppression by further limiting aerobic glycolysis in T cells, impairing their cytotoxic function. Evidence from recent spatial transcriptomics and live-cell imaging suggests that mitochondrial trafficking may be spatially regulated within immune niches, which could explain localized regions of immune suppression within the tumor mass (21).

Therefore, addressing the context dependence of mitochondrial transfer in these immune-responsive tumors could unveil new therapeutic vulnerabilities. Technologies like single-cell metabolomics, immune spatial mapping, and live-cell mitochondrial tracking are poised to deepen our understanding of how mitochondrial trafficking shapes immune landscapes, ultimately informing precision immunotherapy strategies. Despite significant advances in understanding the role of mitochondrial transfer in immune evasion, several critical methodological and experimental challenges remain unresolved, hindering its full translational potential.

In addition to immune-hot tumors, our citation burst analysis revealed that “stem cells” and “tumor microenvironment” are emerging thematic hotspots. Specifically, the increasing focus on MSCs highlights their role as key donors of mitochondria in the TME. MSC-derived mitochondrial transfer has been shown to modulate T cell metabolism, inhibit effector function, and promote immune suppression, suggesting a previously underexplored axis of immune regulation. Similarly, the burst in “tumor microenvironment” emphasizes the growing interest in how local environmental conditions—including stromal composition, immune infiltrates, and hypoxia—shape the direction and immunological outcomes of mitochondrial trafficking. Together, these burst keywords point to new therapeutic strategies that target not just the mitochondrial transfer itself, but also its cellular sources and environmental regulators.

### 4.4 Experimental challenges and methodological pitfalls

Despite the promising findings, the field is hampered by key limitations. First, quantitative metrics of mitochondrial transfer are lacking. Most current studies rely on MitoTracker dyes or fluorescent proteins, which are prone to false-positive interpretations due to dye leakage or fusion artifacts. Flow cytometry-based methods remain underutilized due to technical constraints. Second, functional assays often fail to link mitochondrial uptake to immune consequences. For example, T cell exhaustion markers (e.g., PD-1, TOX, TIM-3) are rarely integrated into mitochondrial transfer experiments, making it difficult to establish causality between organelle exchange and immune dysfunction.

Moreover, few *in vivo* models fully recapitulate the complexity of the human TME, where mitochondrial transfer co-occurs with immunotherapy, hypoxia, and metabolic stress. There is an urgent need for immunocompetent, orthotopic tumor models that enable spatiotemporal tracking of mitochondrial trafficking alongside immune cell dynamics. A comprehensive bibliometric mapping, such as the one we conducted, can visualize current methodological landscapes and highlight underexplored areas that require advanced quantitative or *in vivo* approaches.

In addition to these experimental limitations, our bibliometric study has intrinsic constraints. One limitation lies in the specificity-oriented search strategy. While our query was deliberately designed to focus on mitochondrial transfer events with direct immunological relevance—thereby excluding broader but less specific terms such as “extracellular vesicles” or “organelle transfer”—this approach may have inadvertently omitted relevant studies addressing mitochondrial dynamics in broader biological contexts. Furthermore, the use of a single database (WoSCC) ensured methodological consistency and compatibility with bibliometric tools but may have limited the inclusion of studies indexed only in PubMed, Embase, or Scopus. As a result, some mechanistic or translational studies published in specialized biomedical journals may not have been captured. Future bibliometric efforts should consider expanding database coverage and search terminologies to validate and extend the present findings. While the role of mitochondrial transfer in tumor immune evasion is gaining increasing attention, pharmacological strategies targeting this process remain largely unexplored. Currently, no clinically validated inhibitors of mitochondrial transfer are available, and existing studies have primarily focused on descriptive mechanisms. Future research should aim to identify specific molecular regulators—such as MIRO1, Rho-GTPases, or mitofusins—and assess their therapeutic potential through rigorous *in vivo* validation (4, 22).

### 4.5 Therapeutic potential and clinical translation

Given its multifaceted impact, targeting mitochondrial transfer holds therapeutic potential. Strategies include blocking TNT formation (e.g., via actin polymerization inhibitors), inhibiting mitochondrial trafficking proteins such as Miro1, or modulating TNT-inducing cytokines (e.g., IL-6, TNF-α) (21). Significantly, our citation burst analysis revealed emerging research attention toward “expression” (potentially of regulatory proteins), “stem cells” (as key mitochondrial donors/recipients), and the “tumor microenvironment” in recent years, all of which are pertinent to identifying new therapeutic targets and strategies. Another avenue is immunometabolic reprogramming, such as using metabolic adjuvants (e.g., metformin, 2-DG) to protect T cells from mitochondrial reprogramming by tumor cells.

From a biomarker standpoint, mitochondrial transfer rates or signatures (e.g., upregulation of Miro1, presence of hybrid mitochondria in T cells) could serve as indicators of ICB resistance or early immune exhaustion. These may guide patient stratification in immunotherapy and inform combination strategies. Our bibliometric analysis aimed to identify future research directions for therapeutic exploitation, and these translational aspects are key among them. To overcome these limitations, recent technological innovations are paving the way for unprecedented insights into mitochondrial transfer dynamics.

### 4.6 Integration with future technologies

Recent advances in single-cell metabolomics, immune spatial mapping, and AI-based trajectory inference offer new tools to dissect mitochondrial transfer *in vivo*. For instance, Zhang et al. used mito-GFP and scRNA-seq to identify exhausted T cells with exogenous mitochondrial uptake, correlating with suppressed IFN-γ expression (10). Integration of such multi-omics platforms will be crucial for mechanistic deconvolution.

Furthermore, CRISPR screens targeting mitochondrial trafficking genes may uncover new regulators, and help prioritize druggable targets. Functional screens under co-culture conditions (e.g., tumor–T cell–stromal triculture) may better simulate real TME conditions. While our bibliometric analysis maps the established and currently trending research landscape, these cutting-edge technologies represent the next frontier, likely to drive the evolution of the field beyond the patterns currently visible and address some of the existing methodological limitations.

### 4.7 Clinical implications and translational opportunities

The therapeutic targeting of mitochondrial transfer represents a frontier in immuno-oncology. Strategies that block mitochondrial trafficking pathways, combined with metabolic adjuvants, could enhance T cell persistence and functionality within the TME. Emerging technologies such as single-cell metabolomics, spatial transcriptomics, and immune spatial mapping provide unprecedented resolution to study mitochondrial exchange in real-time. Furthermore, leveraging CRISPR screens to identify key regulators of mitochondrial trafficking may unlock new druggable targets, pushing the boundaries of precision oncology.

Moving forward, integrating high-dimensional immune profiling and organotypic tumor cultures will be essential to recapitulate the complexity of mitochondrial dynamics in immunotherapy contexts. These advances promise not only to deepen mechanistic understanding but also to drive the translation of mitochondrial transfer inhibition into clinical applications.

Translationally, mitochondrial transfer rates or signatures—such as Miro1 upregulation or the presence of hybrid mitochondria in T cells—could serve as indicators of ICB resistance or early immune exhaustion. This predictive capacity may allow for patient stratification in immunotherapy, paving the way for combination strategies that target mitochondrial trafficking alongside ICB. Early-phase clinical trials exploring mitochondrial metabolism modulators (e.g., metformin, 2-DG) have shown potential in enhancing T cell survival, further supporting the viability of this therapeutic avenue.

Thus, a deeper mechanistic understanding, coupled with robust preclinical models, is paramount for the successful translation of mitochondrial transfer inhibition into viable cancer therapies. Future research should prioritize identifying key mitochondrial transfer mediators and validating their roles in immune evasion across various cancer types.

### 4.8 Analytical synthesis and proposed hypothesis

While descriptive studies have established the presence of mitochondrial transfer in tumor settings, recent evidence by Zhang et al. (10) offers a new mechanistic understanding: tumor cells can actively transfer mitochondria into CD8^+^ T cells in human lung cancer and melanoma tissues. These transferred mitochondria evade mitophagy, accumulate in T cells, and cause oxidative stress, leading to T cell senescence and functional exhaustion. Clinically, the presence of tumor-derived mitochondrial DNA in TILs correlates with poor response to anti–PD-1 immunotherapy (10).

Based on these findings, we propose a testable mechanistic model: mitochondrial donation from tumor to T cells depletes host cytotoxic capacity via redox-mediated mitochondrial dysfunction, promoting immune checkpoint resistance. This model reframes mitochondrial transfer not as passive signaling but as a direct metabolic sabotage strategy. Future studies should apply mitochondrial tracking in spatial transcriptomic contexts and evaluate whether combined targeting of mitochondrial trafficking and PD-1 pathways can restore T cell bioenergetics and anti-tumor efficacy.

## 5 Conclusion

This study presents a comprehensive bibliometric analysis of the rapidly expanding field of mitochondrial transfer in tumor immune evasion. By integrating co-occurrence mapping, burst keyword detection, timeline clustering, and co-citation networks, we delineated the conceptual structure and dynamic evolution of the field. Our findings indicate that research has progressively shifted from early descriptions of mitochondrial dynamics and TNTs to mechanistic investigations into their immunological and therapeutic relevance.

Notably, hematologic malignancies such as AML and solid tumors like breast cancer have emerged as dominant models for mitochondrial trafficking studies, particularly in relation to chemoresistance and immune modulation. However, critical gaps remain in our understanding of how mitochondrial transfer influences immune surveillance in immunotherapy-relevant tumors such as melanoma and non-small cell lung cancer.

The field is constrained by methodological limitations, including the lack of *in vivo* imaging techniques and insufficient functional assays in immunological contexts. Nonetheless, the growing interest in targeting mitochondrial trafficking—via molecular regulators such as Miro1 or TNT inhibitors—offers promising translational potential.

We propose that mitochondrial transfer should be considered a functional hallmark of immune evasion in cancer, and future studies should aim to integrate organelle imaging, metabolic profiling, and immune phenotyping to elucidate its role in tumor progression and therapeutic resistance.
